# Expression of the embryonic stem cell marker SOX2 in early-stage breast carcinoma

**DOI:** 10.1186/1471-2407-11-42

**Published:** 2011-01-28

**Authors:** Claudia Lengerke, Tanja Fehm, Ralf Kurth, Hans Neubauer, Veit Scheble, Friederike Müller, Friederike Schneider, Karen Petersen, Diethelm Wallwiener, Lothar Kanz, Falko Fend, Sven Perner, Petra M Bareiss, Annette Staebler

**Affiliations:** 1 University of Tuebingen Medical Center II, Otfried-Mueller-Strasse 10, 72076 Tuebingen, Germany; 2 University of Tuebingen Women's Hospital, Calwerstrasse 7, 72076 Tuebingen, Germany; 3 University of Tuebingen Institute of Pathology, Liebermeisterstrasse 8, 72076 Tuebingen, Germany

## Abstract

**Background:**

The SRY-related HMG-box family of transcription factors member *SOX2 *has been mainly studied in embryonic stem cells as well as early foregut and neural development. More recently, SOX2 was shown to participate in reprogramming of adult somatic cells to a pluripotent stem cell state and implicated in tumorigenesis in various organs. In breast cancer, SOX2 expression was reported as a feature of basal-like tumors. In this study, we assessed SOX2 expression in 95 primary tumors of postmenopausal breast cancer patients.

**Methods:**

Samples from 95 patients diagnosed and treated at the University of Tuebingen Institute of Pathology and Women's Hospital were analyzed by immunohistochemistry for SOX2 expression in the primary tumor samples and in corresponding lymph node metastasis, where present. Furthermore, SOX2 amplification status was assessed by FISH in representative samples. In addition, eighteen fresh frozen samples were analyzed for *SOX2*, *NANOG *and *OCT4 *gene expression by real-time PCR.

**Results:**

SOX2 expression was detected in 28% of invasive breast carcinoma as well as in 44% of ductal carcinoma in situ (DCIS) lesions. A score of SOX2 expression (score 0 to 3) was defined in order to distinguish SOX2 negative (score 0) from SOX2 positive samples (score 1-3) and among latter the subgroup of SOX2 high expressors (score 3 > 50% positive cells). Overall, the incidence of SOX2 expression (score 1-3) was higher than previously reported in a cohort of lymph node negative patients (28% versus 16.7%). SOX2 expression was detected across different breast cancer subtypes and did not correlate with tumor grading. However, high SOX2 expression (score 3) was associated with larger tumor size (p = 0.047) and positive lymph node status (0.018). Corresponding metastatic lymph nodes showed higher SOX2 expression and were significantly more often SOX2 positive than primary tumors (p = 0.0432).

**Conclusions:**

In this report, we show that the embryonic stem cell factor SOX2 is expressed in a variety of early stage postmenopausal breast carcinomas and metastatic lymph nodes. Our data suggest that SOX2 plays an early role in breast carcinogenesis and high expression may promote metastatic potential. Further studies are needed to explore whether SOX2 can predict metastatic potential at an early tumor stage.

## Background

Pluripotency-associated transcription factors like *NANOG*, *SOX2 *and *OCT4 *are known as regulators of cellular identity in embryonic stem cells and more recently have been identified in tumors of various origins. Consistent with their role in sustaining stemness of embryonic stem cells, pluripotency-related factors have been suggested to be expressed with higher frequency in tumors displaying lower degrees of differentiation [1].

In the current study, breast tumor samples were examined for expression of SOX2 (short for Sex determing Region Y - box 2), a High Mobility Group (HMG) domain transcription factor located at chromosome 3q26.33 and member of the SRY-related HMG-box (SOX) family of transcription factors [2]. SOX proteins play critical roles during organogenesis and in the embryonic development of several tissues. Their expression displays a restricted spatial-temporal pattern. For example, overexpression of Sox2 in mouse neural stem cells blocks their differentiation, and conversely, depletion of Sox2 in neural stem cells causes their premature exit from the cell cycle and respectively differentiation into neurons [3,4]. In the foregut, Sox2 is a key regulator of embryonic development and expression is found in all endodermal cells of the undivided foregut. During bronchogenesis in the developing lung, Sox2 is precisely regulated and forced overexpression of Sox2 leads to a block of airway branching [5].

Consistent with the hypothesis that stemness and embryonic pathways may reactivate during oncogenesis, SOX family members have been found to be deregulated in a variety of tumors [4]. SOX2 was detected as an immunogenic antigen in a significant percentage of small cell lung cancer patients [6] and meningeoma patients [7]. In the pancreas, SOX2 expression has been involved in invasion and metastasis of pancreatic intraepithelial neoplasia [8]. Furthermore, SOX2 was also shown to be expressed in gastric [9] and prostate cancers [10] and more recently, was identified as a lineage-survival oncogene in squamous cell carcinomas of the lung [11,12]. However, the significance of SOX2 expression and its role in different cancers requires further research since the transcriptional activity of SOX proteins depends on the recruitment of protein partners and thus profound functional differences may occur in distinct tissues of origin [13].

To our knowledge, there is no data reporting a role of SOX2 in breast organogenesis or function. Adult healthy breast tissue does not show significant SOX2 expression [14]. However, SOX2 expression was detected in a subgroup of patients with breast tumors [15], supporting the notion that in the breast, activation of SOX2 is part of the malignant progression [14,15]. An active role for SOX2 during mammary tumorigenesis is further supported by data collected in breast cancer cell lines, where SOX2 drives cell proliferation and *in vivo *tumorigenesis, partially by facilitating the G_1_/S transition and regulating, in concert with β-catenin, the expression of downstream effector genes such as *CCND1 *[14,15].

In this report, we analyze the expression of SOX2 in a cohort of 95 sporadic postmenopausal early-breast cancers with respect to clinicopathological factors.

## Methods

### Tumor Samples

We analyzed a group of 86 sporadic invasive early-stage breast carcinomas and nine ductal in situ carcinoma (DCIS) diagnosed and treated at the Institute of Pathology and respectively the Women's University Hospital Tuebingen. All tissue samples were derived from a series of consecutive cases at the Department of Pathology analyzing the differences of clinicopathological factors between screening-carcinomas and carcinomas detected outside the screening programme in the same age group. The age of patients ranged therefore from 50 to 69 years and all were diagnosed between March 31^st ^2008 and January 19^th ^2009. From this group a randomly selected subset of 86 cases with available paraffin material was included in this retrospective study. Breast cancer subtypes were defined by immunohistochemistry profiles as previously described [16]. Further clinicopathological characteristics of the cohort are summarized in Table 2. Furthermore, fresh frozen tissue samples were collected prospectively from eighteen patients undergoing diagnosis and treatment in 2009 and 2010 at the Women's University Hospital Tuebingen and used for gene expression analysis as described below.

The study was approved by the institutional Ethics Review Board of the University Hospital Tuebingen.

### Human pluripotent stem cells cultures

The human induced pluripotent stem cell line hFib2-iPS5 kindly provided by George Q. Daley and In-Hyun Park, Children's Hospital Boston [17], was used and grown in undifferentiated state according to previously published protocols [17,18].

### Immunohistochemistry

**Figure 1:**
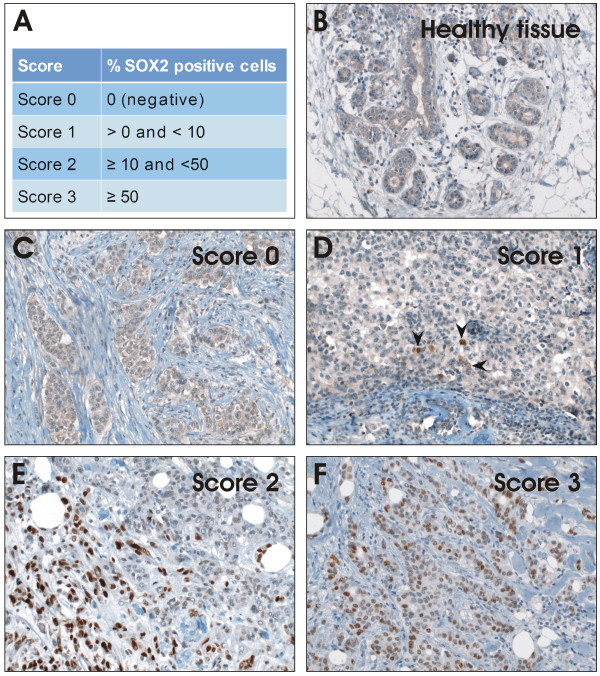
**Immunohistochemical staining of SOX2 shows different expression levels in early-stage breast carcinoma samples. **(A) Classification of SOX2 expression in different scores. (B) Staining of normal breast tissue as control. (C) Breast tumor tissue that shows no positive staining for SOX2 are part of Score 0. (D) Tumor samples with > 0% and < 10% are referred to Score 1. (E) Score 2 samples show ≥ 10% and < 50% positive stained cells. (F) Samples demonstrating ≥ 50% positive cells belong to Score 3. Pictures were taken with 200X magnification

Immunohistochemistry was performed with the Ventana Discovery automated immunostaining system (Ventana Medical Systems, Tucson, AZ, USA), using Ventana reagents. Paraffin sections (5 μm) were mounted on superfrost slides, deparaffinized in inorganic buffer, and pretreated with EDTA-based buffer (pH 8.4). Primary antibody (polyclonal goat anti-human SOX2 antibody, AF2018, R&D systems, dilution 1:40, heat induced epitope retrieval (HIER)) was applied to assess for SOX2 protein expression status. Dilution was performed with Ventana diluent. Bound antibody was visualized using a biotinylated detection kit based on diaminobenzidine and horseradish peroxidase (DABMap-kit, Ventana). Slides were counterstained with hematoxylin and Blueing Reagent (Ventana). Subsequently, sections were washed, dehydrated in a graded alcohol series and covered with Cytoseal. Only nuclear staining was considered positive and scored by a pathologist according to published criteria using a semiquantitative score: score 0: no positive cells, score 1: >0 to 10%, score 2 ≥ 10%, score 3 ≥ 50% [19] (Figure 1A-E). As positive controls were used samples of squamous cell carcinoma of the lung [20].

### *SOX2 *amplification fluorescence in-situ hybridization assay

To assess for *SOX2 *amplification status at the chromosomal level, we applied the same two-color interphase FISH assay as described by Bass et al. [20]. Briefly, a probe spanning the locus 3q26.33 (BAC clone CTD-2348H10) was applied to detect *SOX2 *copy number status and was compared to a reference probe hybridizing to 3p22.3-3p22.2 (BAC clone RP11-286G5) (both clones were purchased from Invitrogen, Carlsbad, CA, USA). The target probe was labelled with biotin and detected with a streptavidin-conjugated red fluorochrome (SAV-Alexa Fluor-594, Invitrogen). The reference probe was labelled with digoxigenin and detected via an anti-digoxigenin-conjugated green fluorochrome (FITC, Roche, Basel, Switzerland).

Assessment of the *SOX2 *amplification status was performed semiquantitatively by comparing the number of red signals (*SOX2 *target region, respectively) to the number of corresponding green signals (reference region). A non-amplified nucleus showed one red target signal for every corresponding green reference signal, with a red/green ratio of 1:1. TMA slides were analyzed under a 63x oil immersion objective using a fluorescence microscope (Zeiss, Jena, Gemany) equipped with appropriate filters. At least 100 nuclei per case were assessed. Cases were included into the analysis if there was at least one core assessable.

### Gene expression analysis

Total RNA from fresh frozen tissue samples was isolated using RNA isolation kit from Qiagen according to the manufacturer's instructions, including Dnase I treatment to remove contaminating genomic DNA (Invitrogen). Purified RNA samples were used for RT reaction containing oligo d(pT)18 primers and Superscript II RT enzyme (Invitrogen) according to the supplier's protocol. The synthesis of cDNA was carried out for 50 min at 42°C followed by 10 min at 70°C to inactivate the RT enzyme. The amplification of *SOX2*, *NANOG*, *OCT4*, and glyceraldehyde-3-phosphate dehydrogenase (*GAPDH*) genes in the subsequent RT-PCR was achieved with the following primer pairs (Sigma) and probes (Roche): For *SOX2 *(75 bp): 5'-ttgctgcctctttaagactagga-3', 5'-ctggggctcaaacttctctc-3', and Probe #35; for *NANOG *(103 bp): 5'-atgcctcacacggagactgt-3', 5'-aagtgggttgtttgcctttg-3', and Probe #31; for *OCT4 *(114 bp): 5'-agcaaaacccggaggagt-3', 5'-ccacatcggcctgtgtatatc-3', and Probe #35; for *GAPDH *(66 bp): 5'-agccacatcgctcagacac-3', 5'-gcccaatacgaccaaatcc-3', and Probe #60. The PCR reaction mixture was incubated at 95°C for 10 min, followed by 35 cycles of 95°C for 15 s, 60°C for 30 s and 72°C for 1 s. As positive control we used RNA purified from undifferentiated human induced pluripotent stem cells. The amplification of *SOX2OT*, *ALX4 *and *ACTIN *genes was achieved with SYBR-Green (Eurogentec) and the following primer pairs: For *SOX2OT *(76 bp): 5'-tccatggaatgaatgaaatgtt-3', 5'-cagcctccaagacctagcc-3'; for *ALX4 *(99 bp): 5'-tggccatgaggacagacc-3', 5'-gctgcatctgcccaaaac-3'; for *ACTIN *(86 bp): 5'-agtcctgtggcatccacgaaacta-3', 5'-cactgtgttggcgtacaggtcttt-3'. The PCR reaction mixture was incubated at 95°C for 10 min, followed by 40 cycles of 95°C for 15 s, 60°C for 1 min and a melting curve 40 - 95 °C for 1 s. RNA purified from undifferentiated human induced pluripotent stem cells was used as a positive control.

### Statistical Analysis

To test associations between categorical variables, we used the Chi square and Fisher's exact test. Values of p < 0.05 were considered significant. All tests were two-tailed and 95% confidence intervals were adopted. The analyses were carried out using the SPSS 12.0 for Windows statistical program (SPSS Inc., Chicago, IL).

## Results

### SOX2 expression in early-stage breast cancer

95 patients yielded material that could be analyzed immunhistochemically for SOX2 expression. Human squamous lung cancer samples were used as positive controls for immunohistochemical detection of SOX2 expression [20]. Four expression scores were defined in order to distinguish SOX2 negative and positive samples, and among latter the subgroup of SOX2 high expressors (Figure 1A). Considering cases with any SOX2 expressing cells as positive (score 0 vs. score 1-3) nuclear SOX2 expression was detected in 24 out of 86 analyzed samples of invasive breast carcinoma and 4 out of 9 DCIS (Table 1). Of note, while numbers of positive cells were highly variable, the expression showed comparable strong intensity among samples and was mostly restricted to the nucleus, as previously reported in embryonic stem cells. Thus, intensity and localization of the positive signal was not introduced as a variable in the applied scoring system. Representative stainings for tumors belonging to each score group are shown in comparison to healthy breast tissue (Figure 1).

**Table 1:** SOX2 expression in primary carcinoma (DCIS and invasive carcinoma) and lymph node samples

	*N total*	*Score 0*	*Score 1*	*Score 2*	*Score 3*
***DCIS***	9	5 (55.6%)	1 (11.1%)	2 (22.2%)	1 (11.1%)

***Invasive Carcinoma***	86	62 (72%)	11 (12.8%)	5 (5.8%)	8 (9.3%)

***Lymph nodes***	18	9 (50%)	4 (22.2%)	1 (5.5%)	4 (22.2%)

DCIS: ductal carcinoma in situ

### Correlation between SOX2 expression and clinicopathological characteristics

**Figure 2:**
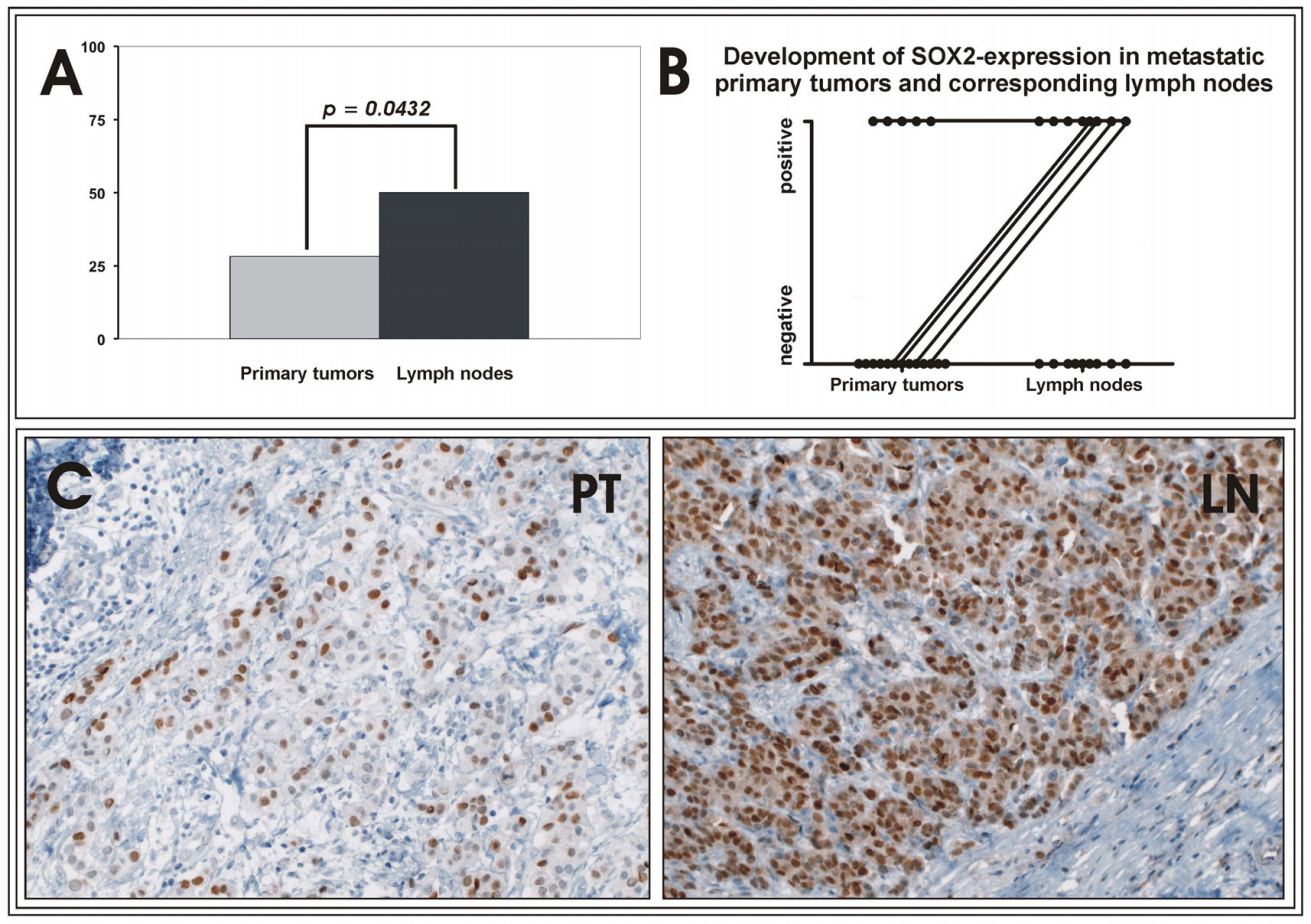
**SOX2 expression in primary tumors and corresponding metastatic lymph nodes. **(A) SOX2 is expressed in a higher percentage of metastatic lymph nodes as compared to primary tumors; (B) Development of SOX2 expression in lymph nodes in comparison to the corresponding metastatic primary tumor investigated in 18 samples. Positive SOX2 expression was detected in lymph node metastases originating from both SOX2 positive or negative primary tumors while no loss of SOX2 positivity was observed during progression from primary tumor to metastasis (C) Representative immunohistochemical staining of SOX2 in a primary tumor and the corresponding lymph node. Pictures were taken with 200X magnification.

Our comparative study of SOX2 negative and positive tumors did not show significant correlations between the SOX2 expression status and other tumor parameters such as grading, breast cancer subtype, hormone receptor or HER2 expression, or presence of lymphangiosis. A trend to larger tumor size (p = 0.073) and to a histology other than of ductal or lobular type was noted in SOX2 expressors (p = 0.053; Table 2). Interestingly, if high SOX2 expressors (score 3) were analyzed separately and compared to the rest of the group (score 0 to 2), they displayed significantly more often lymph-node metastases (p = 0.018) and larger primary tumors (p = 0.047; Figure 2; Table 3). Although larger numbers are required to analyze expression in DCIS, our data indicate that SOX2 is expressed already aberrantly in DCIS and therefore may be an early event in disease progression.

**Table 2:** Correlation of SOX2 score and clinicopathological parameters

		*SOX2*		
			
	*N total*	*Negative Score 0*	*Positive Score 1-3*	*p-value*
***Tumor size***				
pT1	59	46 (78%)	13 (22%)	*0.073*
pT2 to pT4	27	16 (59%)	11 (41%)	

***Nodal status***				
Node-negative	62	44 (71%)	18 (29%)	*0.789*
Node-positive	23	17 (74%)	6 (26%)	

***Histology***				
Ductal	76	49 (73%)	18 (27%)	
Lobular	11	10 (91%)	1 (10%)	*0.053*
Others	8	5 (38%)	5 (63%)	

***Grading***				
I-II	60	45 (75%)	15 (25%)	*0.361*
III	26	17 (65%)	9 (35%)	

***Lymphovasc. inv.***				
Negative	63	48 (76%)	15 (24%)	*0.161*
Positive	23	14 (61%)	9 (39%)	

***ER status***				
Negative	17	12 (71%)	5 (29%)	*0.877*
Positive	69	50 (73%)	19 (28%)	

***PR status***				
Negative	22	16 (73%)	6 (27%)	*0.939*
Positive	64	46 (72%)	18 (28%)	

***HER2***				
Negative	75	55 (73%)	20 (27%)	*0.503*
Positive	11	7 (64%)	4 (36%)	

***Subtype***				
Luminal A/B *	73	53 (73%)	20 (27%)	
HER2 subtype	6	4 (67%)	2 (33%)	*0.952*
Triple negative	7	5 (71%)	2 (29%)	

*Luminal A tumors are defined as tumors with expression of one or both hormone receptors without overexpression of Her2; Luminal B tumors express one or both hormone receptors and show also Her2 overexpression.

**Table 3:** SOX2 high expressors (Score 3) *versus *negative or low (Score 0-2)

	*p-value*
***Tumor size***	***0.047***

***Nodal Status***	***0.018***

***Histology***	*0.272*

***Grading***	*0.252*

***Lymphovascular Invasiveness***	*0.470*

***ER Status***	*0.140*

***PR Status***	*0.373*

***Subtype***	*0.456*

ER: Estrogen Receptor; PR: Progesteron Receptor.

### SOX2 FISH and gene expression analysis in primary tumors

**Figure 3:**
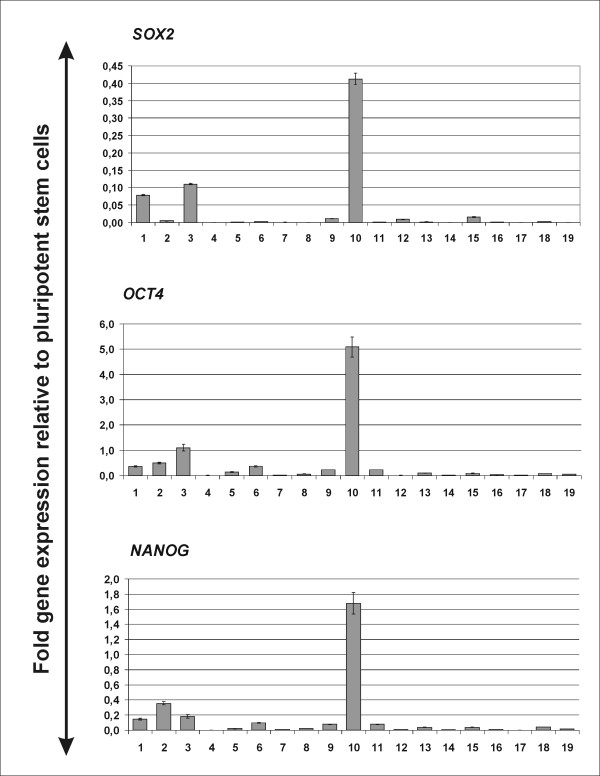
**Gene expression of *SOX2*, *NANOG *and *OCT4 *in different tumor samples shows clustering of embryonic factors in certain tumors. **Real-time PCR for *SOX2*, *NANOG *and *OCT4 *was performed on isolated RNA from tumor tissue. RNA from undifferentiated human pluripotent stem cells was used as a control. Shown are fold relative gene expression levels in comparison to undifferentiated pluripotent stem cells. Experiments have been performed in triplicates: error bars depict standard deviations.

Eleven SOX2 positive samples (7 belonging to expression score 3 and 4 of score 2), 4 SOX2 negative samples as well as 3 lymph-node samples showing high SOX2 expression (score 3) were analyzed by FISH to explore whether aberrant SOX2 expression is a result of gene amplification as previously reported in other carcinomas [20]. Surprisingly, with the exception of one case of low level amplification documented in a score 3 primary tumor, unlike reported in other tumors, the majority of analyzed samples did not show SOX2 gene amplifications, suggesting that at least in part of the breast carcinomas expressing SOX2, the aberrant gene expression is driven by other mechanisms. To explore whether *SOX2 *induction is part of a more general reactivation of embryonic genes, we assessed co-expression of *NANOG *and *OCT4 *in the same tumors by performing real-time PCR analysis and using human pluripotent stem cells as positive controls [17]. Among fresh frozen samples collected prospectively from n = 18 patients we observed various degrees of *SOX2 *gene expression (Figure 3), confirming our immunohistochemical data. However, samples showing more pronounced *SOX2 *expression levels (sample 1, 3 and 10) displayed substantial co-expression of *OCT4 *and *NANOG *(Figure 3). Furthermore, co-expression with the previously described *SOX *interacting gene *ALX4 *as well as with the SOX2-overlapping transcript (*SOX2OT*) could be documented in *SOX2-*expressing samples (Additional file 1, Figure S1) [21,22].

### Higher SOX2 expression in metastatic lymph nodes

To further dissect whether SOX2 plays a role in development of metastases, we analyzed SOX2 expression in metastatic lymph nodes. 23 out of 86 patients with invasive primary tumors showed lymph node metastases, out of which 18 yielded material that could be analyzed for SOX2 expression (Table 1, Figure 2). The remaining 5 cases only presented with positive sentinel lymph-nodes, which had been completely analyzed in step sections, and thus no material for additional SOX2 analysis was available. As expected, SOX2 expression was detected in all lymph-nodes from SOX2 positive primary tumors (Figure 2A-B). Interestingly, SOX2 was additionally detected in lymph-nodes derived from primary tumors devoid of SOX2 expression (Figure 2B and 2D) while no case could be detected where SOX2 was expressed only in primary but not in metastatic cells. Thus, the frequency of SOX2 positive samples in lymph nodes was significantly higher than those of primary tumors (p = 0.0432; Figure 2B). Of note, 3 out of 9 positive lymph nodes showed very high expression in nearly all tumor cells, while such high expression was only observed in 1 out of 24 positive primary tumors.

## Discussion

SOX2, NANOG and OCT3/4 form the core of the self-renewal transcription network in embryonic stem cells. They physically interact with each other forming large protein complexes [20], and furthermore are transcriptionally interconnected and co-occupy promoters of numerous target genes [23-26]. On a functional level, selective downregulation of one of these factors induces embryonic stem cell differentiation and exit from the pluripotent stem cell state. More recently, combinatorial overexpression of OCT4, SOX2, NANOG and others was shown to reprogram several types of adult somatic cells to a pluripotent stem cell like state [17,27,28]. In these experiments, cells were reprogrammed fully or only partially [29] possibly through heterogeneous exposure to reprogramming factors. It is tempting to speculate that acquisition or overexpression of individual factors (i.e. by chromosomal gain, as described in some tumors), can promote tumorigenesis by processes resembling partial reprogramming [17,29].

In our study we have focused on SOX2, a member of the SOX (SRY-related HMG-box) family of transcription factors. SOX proteins are expressed during early embryogenesis and play important roles in embryonic and extra-embryonic cell types [30]. To our knowledge there is no data indicating a specific role of SOX2 during breast tissue development and as far no relevant SOX2 expression could be detected in healthy human breast specimens [14]. However, SOX2 expression was described in a smaller percentage (16.7%) of lymph-node negative breast carcinomas, suggesting a role in breast tumorigenesis.

Breast cancer is a heterogeneous disorder presenting in a variety of pathological entities and clinical manifestation ranges. Based on molecular profiling and gene expression signatures, five groups of breast cancers with distinct prognostic and predictive significance have been identified: basal-like, luminal A and B, HER2+ and normal breast-like carcinomas [31]. Of these, the most malignant phenotype is shown by the basal-like cancers encompassing high grade tumors negative for estrogen receptor (ER) or HER2 expression, which are associated with a tendency to visceral metastasis especially to the lung and the brain and which also have the most reproducible gene expression pattern across different studies and technical platforms [32-35]. Among sporadic cancers, basal-like tumors are showing most genetic and phenotypic similarities to the aggressive tumors arising in BRCA1 germ line mutation carriers [32-34,36-39]. Consistently, 3q gains are most frequently observed in tumors arising in BRCA1 mutation carriers [40,41] and, among sporadic cancers, seen with highest incidence in basal-like tumors (20% of cases, in comparison to 10% of luminal tumors [42]).

In squamous lung and esophageal cancers, aberrant SOX2 expression was linked to the genomic amplification of its chromosomal location on chromosome 3q26.33. 3q copy number gains are a common event in breast cancers and have been implicated as an independent predictor of poor prognosis in node-negative breast cancers [43]. A previous immunohistochemical study performed in a cohort of lymph-node negative patients observed predominant SOX2 expression in tumors with basal-like phenotype [15], consistent with the pattern described for 3q chromosomal gain. In our study on a cohort of postmenopausal patients displaying both negative and positive lymphonodal status we could confirm the aberrant expression of SOX2 in breast cancer. Interestingly, we assessed a higher overall incidence of SOX2 expression than reported by the previous study (ca. 28%). This could be due to technical details and different sensitivities of immunohistochemistry protocols, although similar methods and the same cut-off definition for SOX2 positivity were applied. A more plausible explanation is that inclusion of lymph-node positive patients in our cohort may have enriched for samples expressing SOX2. In contrast to the previous study, we could not verify the correlation between SOX2 expression status and breast cancer with basal-like features that is triple negativity for hormone receptors and HER2 [16], although associations with other parameters reflecting tumor aggressiveness such as tumor size and positive lymphonodal status were observed. Taken together, these results suggest that aberrant SOX2 expression plays a broader role in breast cancer pathogenesis, exerting effects also outside of the subgroup of triple negative tumors. However, it is possible that SOX2 expression is indeed particularly enhanced in triple negative tumors and that our analysis in a cohort of postmenopausal patients, where this molecular subtype is underrepresented, failed to detect this correlation because of low numbers. While some (especially the 3q positive basal-like tumors) may acquire SOX2 as a result of gains in specific chromosomal regions, SOX2 expression may be also induced by other upstream mechanisms inducing a general reactivation of an embryonic genetic program. In support of this hypothesis, FISH analysis performed in representative primary tumor and lymph node samples with high SOX2 expression in most cases did not show amplification of the SOX2 locus. Furthermore, SOX2 was detected to express in concert with other pluripotency factors in a cohort of 18 patients analyzed by real-time PCR analysis where high SOX2 expressing samples were found to also express higher levels of OCT4 and NANOG (Figure 3).

*In vitro *and *in vivo *tumorigenesis studies performed with breast cancer cell lines link SOX2 expression to early events in tumor development and potentially to tumor invasiveness [14]. Similar properties have been shown in lung squamous cell carcinoma lines where *in vitro *studies suggest SOX2-mediated induction of cell proliferation and anchorage independent growth [11,12,14]. Although SOX2 functions differ between organ systems since transcriptional activation is influenced by the recruitment of tissue-specific transcription factors [44], SOX2-mediated induction of tumor invasiveness may be a common theme in different tumor entities [45]. To further explore these findings in breast cancer, we performed correlations between SOX2 expression levels and lymphonodal status and explored expression of SOX2 in metastatic lymph nodes as well as in earlier disease stages such as ductal carcinoma in situ. Overall, our immunohistochemical study provides evidence supporting an early role of SOX2 during disease pathogenesis, since similar expression levels were found in DCIS and early-stage invasive tumors. However, expression was significantly higher in metastatic lymph nodes supporting the notion that SOX2 plays a role in disease invasiveness and progression. Since no primary tumor expressing SOX2 produced a lymph-node metastasis devoid of SOX2 expression and SOX2 positive lymph-nodes showed particularly high expression levels, we suggest that cells displaying SOX2 expression are enriched in metastatic potential and SOX2 plays a specific role in the development of lymph-node metastases. However, further studies are needed to deepen our understanding of SOX2 and other embryonic factors during mammary tumorigenesis and larger numbers of prospectively collected samples should be screened before proposing SOX2 as a predictor of lymphonodal status in breast cancer.

## Conclusions

The embryonic stem cell factor SOX2 is expressed in a variety of early stage postmenopausal breast carcinomas and metastatic lymph nodes. Our data suggest that SOX2 plays an early role in breast carcinogenesis and high expression may promote metastatic potential. Further studies are needed to explore whether SOX2 can predict metastatic potential at an early tumor stage.

## Competing interests

The authors declare that they have no competing interests.

## Authors' contributions

CL, TF, RK, PMB, HN, SP and AS designed the experiments and analyzed the data. PMB, RK, FM, KP and VS performed experiments. FS and AS retrieved the cases from the archives of the Department of Pathology, reviewed the diagnoses and established the database with clinical and pathological parameters. AS and TF performed the statistical analysis. CL,TF, DW, LK, FF and AS designed the research. CL wrote the paper, AS took the pictures, PMB made the figures and tables. All authors contributed to editing the manuscript. All authors have read and approved the final manuscript.

## Acknowledgements and Funding

We thank George Q. Daley, Children's Hospital Boston, Harvard Medical School and Howard Hughes Medical Institute, and In-Hyun Park, Yale School of Medicine for providing the human pluripotent stem cell line hFib2-iPS5. The work of CL was supported by the Max-Eder-Program of the Deutsche Krebshilfe and the Deutsche Forschungsgemeinschaft (SFB773).

## Pre-publication history

The pre-publication history for this paper can be accessed here:

http://www.biomedcentral.com/1471-2407/11/42/prepub

## Supplementary Material

Additional file 1 **Supplementary Figure 1: Gene expression of *SOX2OT and ALX4 in *fresh frozen tumor samples**. Real-time PCR for *SOX2OT *and *ALX4 *was performed on isolated RNA from tumor tissue. RNA from undifferentiated human pluripotent stem cells was used as a control. Shown are fold relative gene expression levels in comparison to undifferentiated pluripotent stem cells. Click here for file
