# Supplementary figures and images for: Expression of the embryonic stem cell marker SOX2 in early-stage breast carcinoma
Source: BMC Cancer. 2011 Jan 28;11:42. doi: 10.1186/1471-2407-11-42 (PMC3038979; doi:10.1186/1471-2407-11-42)

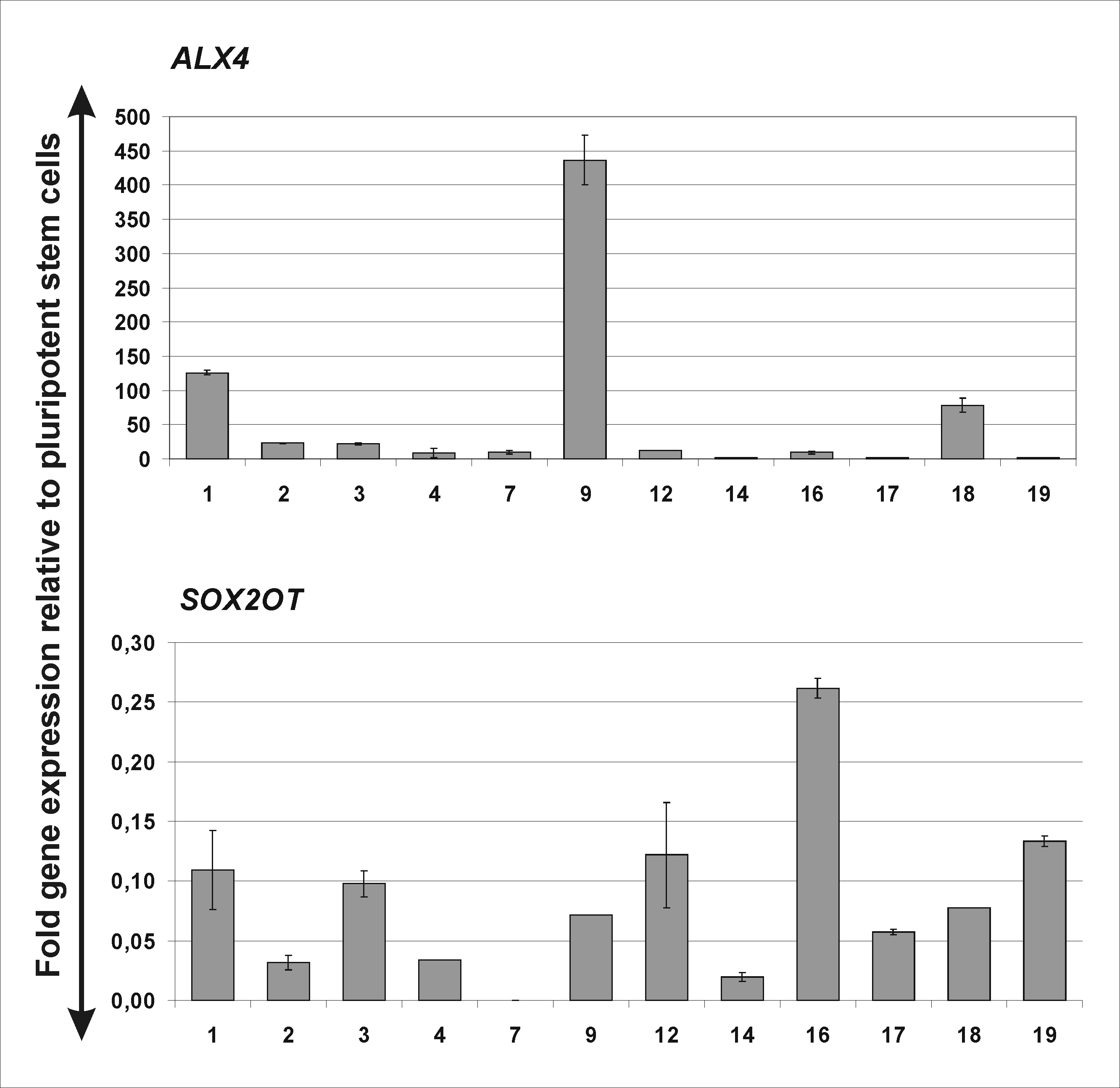

Supplement: Additional file 1 — Supplementary Figure 1: Gene expression of SOX2OT and ALX4 in fresh frozen tumor samples. Real-time PCR for SOX2OT and ALX4 was performed on isolated RNA from tumor tissue. RNA from undifferentiated human pluripotent stem cells was used as a control. Shown are fold relative gene expression levels in comparison to undifferentiated pluripotent stem cells. [file 1471-2407-11-42-S1.TIFF]
